# Preadmission statin use and one-year mortality among patients in intensive care - A cohort study

**DOI:** 10.1186/cc8902

**Published:** 2010-03-09

**Authors:** Steffen Christensen, Reimar W Thomsen, Martin B Johansen, Lars Pedersen, Reinhold Jensen, Kim M Larsen, Anders Larsson, Else Tønnesen, Henrik Toft Sørensen

**Affiliations:** 1 Department of Clinical Epidemiology, Aarhus University Hospital, Olof Palmes alle 43-45, Århus N, 8240, Denmark; 2 Department of Anaesthesiology and Intensive Care, Skejby Hospital, Aarhus University Hospital, Brendstrupgårdsvej 100, Århus N, 8240, Denmark; 3 Department of Anaesthesiology and Intensive Care, Aarhus Hospital, Aarhus University Hospital, Nørrebrogade 44, Århus C, 8000, Denmark; 4 Department of Anaesthesiology and Intensive Care, Aalborg Hospital, Aarhus University Hospital, Hobrovej 18, Ålborg, 9100, Denmark

## Abstract

**Introduction:**

Statins reduce risk of cardiovascular events and have beneficial pleiotropic effects; both may reduce mortality in critically ill patients. We examined whether statin use was associated with risk of death in general intensive care unit (ICU) patients.

**Methods:**

Cohort study of 12,483 critically ill patients > 45 yrs of age with a first-time admission to one of three highly specialized ICUs within the Aarhus University Hospital network, Denmark, between 2001 and 2007. Statin users were identified through population-based prescription databases. We computed cumulative mortality rates 0-30 days and 31-365 days after ICU admission and mortality rate ratios (MRRs), using Cox regression analysis controlling for potential confounding factors (demographics, use of other cardiovascular drugs, comorbidity, markers of social status, diagnosis, and surgery).

**Results:**

1882 (14.3%) ICU patients were current statin users. Statin users had a reduced risk of death within 30 days of ICU admission [users: 22.1% vs. non-users 25.0%; adjusted MRR = 0.76 (95% confidence interval (CI): 0.69 to 0.86)]. Statin users also had a reduced risk of death within one year after admission to the ICU [users: 36.4% vs. non-users 39.9%; adjusted MRR = 0.79 (95% CI: 0.73 to 0.86)]. Reduced risk of death associated with current statin use remained robust in various subanalyses and in an analysis using propensity score matching. Former use of statins and current use of non-statin lipid-lowering drugs were not associated with reduced risk of death.

**Conclusions:**

Preadmission statin use was associated with reduced risk of death following intensive care. The associations seen could be a pharmacological effect of statins, but unmeasured differences in characteristics of statin users and non-users cannot be entirely ruled out.

## Introduction

There is substantial evidence that statins - widely used lipid-lowering drugs - are effective in reducing major cardiovascular events and mortality in patients with arteriosclerotic disease, diabetes and hypercholesterolemia [1-4]. Experimental studies reported evidence that statins may also have anti-inflammatory, anti-thrombotic and immuno-modulating effects independent of lowering lipids, also referred to as pleiotropic properties [5,6]. The pleiotropic properties may differ between individual statins, in particular between lipophilic and hydrophilic statins [7-9].

In line with the experimental findings, observational studies have reported a risk reduction [10-12] and profound improvements in the outcome of critical illnesses such as severe infections among statin users [13-17]. Beneficial effects in terms of reduced morbidity and mortality of statins have also been reported among patients with chronic obstructive pulmonary disease, chronic renal failure and in patients undergoing cardiac or major non-cardiac surgery [18-21].

Patients in intensive care units (ICUs) have a high prevalence of cardiovascular diseases, severe infections and thrombotic complications, and almost all ICU patients suffer from the systemic inflammatory response syndrome [22,23]. Statins could thus have beneficial effects on mortality following ICU admission. The three existing studies on this issue reported conflicting results and were limited by small and highly selected study populations such as patients with multiple organ dysfunction syndrome or acute lung injury/adult respiratory distress syndrome (ALI/ARDS), and assessed mortality only while in the hospital [24-26]. As intensive care treatment is common, expensive and often ends in death, any beneficial effect of statins in this setting has major clinical and public health implications.

We conducted a large cohort study, based on more than 12,000 ICU patients, to examine the extent to which preadmission statin use overall and by specific agent was associated with risk of death in the year following ICU admission.

## Materials and methods

### Setting

We conducted this cohort study based on prospectively collected data obtained from medical databases in northern Denmark between 1 January, 2001 and 31 December, 2007. The Danish national health care system provides the entire Danish population with unrestricted access to tax-supported public health services and all critically ill patients receive care in public hospitals [27].

Since 1968 every Danish citizen has received at birth a unique civil registration number from the Danish Civil Registration System. This number permits accurate linkage across all Danish registries [28].

The study population comprised all patients admitted for the first time to an ICU in one of three hospitals within the Aarhus University Hospital network (Aalborg, Aarhus and Skejby Hospitals) [29]. All ICUs are highly specialized multidisciplinary units serving both as primary and referral ICUs. Their patients include those with severe respiratory failure requiring extracorporeal membrane oxygenation and patients undergoing organ transplantation.

### Intensive care data

ICU patients were identified using a research database (Aarhus University Intensive Care Cohort (AUICC)). Data on use of mechanical ventilation, use of renal replacement therapy, dates of ICU admission and discharge, and civil registration numbers for all patients treated in the three ICUs are recorded by ICU physicians as part of standard department clinical practice and are routinely entered into the AUICC. We did not include in the cohort patients who were admitted for planned postoperative observation of less than 24 hours and did not include patients younger than 45 years of age, a group that is rarely prescribed statins [30]. The study cohort totaled 12,483 eligible ICU patients with a first ICU admission during the study period.

### Preadmission use of statins

We used a prescription database covering the entire region since 1998 to identify statin users. The database contain data, transferred electronically from all pharmacies in the region, on types and dosages of all reimbursed drugs prescribed, customers' civil registration numbers and redemption dates [31]. We defined current statin use as at least one filled prescription for statins within 125 days before ICU admission (for details on Anatomical Therapeutic Chemical (ATC) codes see Additional file 1) [31]. The 125-day period allowed us to capture most current statin users, because in Denmark few statin prescriptions are expected to last more than 125 days [17,30]. Among current statin users, we distinguished 'new' and 'long-term' statin users as those who had filled their first statin prescription within 125 days before ICU admission or earlier than 125 days, respectively [32].

Statin users may have been more frequently hospitalized during the exposure defining period before ICU admission than non-users. As no prescriptions are filled during hospitalizations this may lead to misclassification of statin use [33]. We therefore used the Danish National Registry of Patients (NRP) to identify the number of days that study patients were hospitalized within the 125 days before ICU admission (0 days, 1 to 10 days,11 to 25 days, >25 days).

### Other prognostic factors

We identified the main diagnosis for the admission requiring intensive care through the Danish NRP. The NRP covers all hospitalizations in Denmark since 1977 and all out-patient hospital visits since 1995 [34]. We grouped patients into eight disease categories: infectious diseases; endocrinology including diabetes; cardiovascular diseases; respiratory diseases; gastrointestinal and liver diseases; cancer; trauma and poisoning; and others (for details on International Classification of Diseases (ICD) codes, see Additional file 1). We also identified the department that transferred the patient to the ICU (surgical/medical). Using the NRP, we obtained information on surgical procedures in the seven days before ICU admission and classified patients as surgical and medical (no surgery within seven days before ICU admission) [35]. To control for comorbidity, we computed the Charlson comorbidity score based on the entire previous discharge history and defined three comorbidity levels: low (score of 0), medium (1 to 2), and high (≥ 3)) [36]. The index includes 19 major disease categories and has been validated as a predictor of mortality [37,38]. We also retrieved information on alcoholism-related disorders, and prescriptions for disulfiram (ICD and ATC codes are provided in Additional file 1). For the subcohort of patients admitted between 2001 and 2006 we collected data on hemoglobin, white blood cell (WBC) count, platelets, C-reactive protein (CRP) and bilirubin on ICU admission and on the most recent total cholesterol level recorded within six months of ICU admission from laboratory databases. We retrieved prescription data on current use of angiotensin converting enzymes (ACE) inhibitors, beta blockers, and low-dose aspirin, because these drugs may confound studies on clinical effects of statins [18,39], and on current use of non-statin lipid-lowering drugs, because these drugs are given on almost the same indications as statins but lack the pleiotropic effects (e.g. niacin, bile acid-binding resin and fibric acid derivatives). As a measure of social status, we obtained data on marital status at the time of ICU admission from the Civil Registration System [28].

### Mortality data

We accessed data from the Civil Registration System, which contains complete information for the entire Danish population on migration and changes in vital status, including exact date of death, and is updated on a daily basis [28].

### Statistical analysis

Follow up began on the date of first-time ICU admission and continued until death, migration, 365 days after ICU admission or 31 December 2008, whichever came first. We computed Kaplan Meier curves and life table estimates for mortality at 30 days and one year for the following variables: preadmission statin use; primary diagnosis; age group; gender; department (medical/surgical); level of Charlson score; alcoholism-related diseases; surgery within seven days (yes/no); need for mechanical ventilation or renal replacement therapy; current use of ACE inhibitors, low-dose aspirin or beta blockers; marital status (married, divorced, widowed, never married, or unknown).

We used Cox proportional hazards regression to compute mortality rate ratios (MRRs) for statin users compared with non-users, controlling for all covariates in Tables 1 and 2, except mechanical ventilation, dialysis, and laboratory data because we considered these potential effects of the intervention [40]. We did separate analyses for subgroups defined according to admitting department, surgery, presence of mechanical ventilation, renal replacement therapy, and for former, new, and long-term statin users. As the pleiotropic effects may vary between types of statins we also did a separate analysis for users of the lipophilic simvastatin, and the hydrophilic atorvastatin and pravastatin and used Wald statistics to compute *P *values for the difference in MRR between types of statins.

**Table 1:** Baseline characteristics by preadmission statin use among 12,483 ICU patients, Aarhus University Hospital, Denmark 2001 to 2007

	Statin use n (%)	No statin use n (%)
**Overall**	1882 (14.3%)	10,601 (85.7%)
**Age group (years)**		
45-60	373 (19.8%)	3725 (35.1%)
61-75	1095 (58.2%)	4421 (41.7%)
76+	414 (22.0%)	2455 (23.2%)
**Gender**		
Male	1193 (63.4%)	6085 (57.4%)
Female	689 (36.6%)	4516 (42.6%)
Comorbidity score*		
Low	283 (15.0%)	3563 (33.6%)
Medium	862 (45.8%)	4280 (40.4%)
High	737 (39.2%)	2758 (26.0%)
Comorbidity diagnosis**		
Ischemic heart disease	658 (35.0%)	950 (9.0%)
Congestive heart failure	406 (21.6%)	1113 (10.5%)
Peripheral vascular disease	451 (24.0%)	1050 (9.9%)
Cerebrovascular disease	468 (24.9%)	1393 (13.1%)
COPD	339 (18.0%)	1836 (17.3%)
Diabetes	456 (24.2%)	879 (8.3%)
Cancer	299 (15.9%)	2401 (22.5%)
Renal disease	171 (9.0%)	586 (5.4%)
Alcoholism-related diseases	137 (7.3%)	1305 (12.3%)
**Cardiovascular drug use**		
ACE inhibitors	1003 (53.3%)	1860 (17.6%)
Beta blockers	983 (52.2%)	1706 (16.1%)
Low-dose aspirin	336 (17.9%)	674 (6.4%)
**Marital status**		
Married	1141 (60.6%)	5664 (53.4%)
Divorced	251 (13.3%)	1512 (14.3%)
Widowed	345 (18.3%)	2033 (19.2%)
Never married	139 (7.4%)	1135 (10.7%)
Unknown	6 (0.3%)	257 (2.4%)

*Level of Charlson comorbidity index.

** Patients may have more than one comorbidity.

ACE: angiotensin converting enzyme; COPD: chronic obstructive pulmonary disease; ICU: intensive care unit.

**Table 2:** Characteristics associated with the current hospitalization of 12,483 ICU patients with and without preadmission statin use, Aarhus University Hospital, Denmark, 2001 to 2007

	Statin use n (%)	No statin use n (%)
**Department**		
Medical	771 (41.0%)	4243 (40.0%)
Surgical	1111 (59.0%)	6358 (60.0%)
**Main diagnosis**		
Infections	43 (2.3%)	253 (2.4%)
Cancer	163 (8.7%)	1607 (15.2%)
Diabetes	30 (1.6%)	129 (1.2%)
Cardiovascular	800 (42.5%)	2828 (26.7%)
Respiratory	132 (7.0%)	1049 (9.9%)
Gastrointestinal	163 (8.7%)	1187 (11.2%)
Trauma/poisoning	136 (7.2%)	1383 (13.1%)
Other	415 (22.0%)	2165 (20.4%)
**Surgical status at ICU admission**		
Surgery within 7 days	199 (10.6%)	1347 (12.7%)
**Mechanical ventilation**	1021 (54.3%)	4796 (45.3%)
**Renal replacement therapy**	223 (11.9%)	867 (8.2%)
Laboratory findings, median (IQR)*		
Hemoglobin (ref; female: 7.4-9.6 mmol/l, male:8.4-10.8 mmol/l)	7.10 (6.30-8.20)	7.30 (6.40-8.30)
Creatinine (ref 60-125 μmol/l)	106.5 (80-165)	93 (70-138)
Bilirubin (ref 4-21 mmol/l)	10 (7-16)	12 (8-20)
C-reactive protein (ref <10 mg/l)	80 (15-250)	97 (21-279)
White blood cell count (ref 4.0-11.0 × 10^9^/l)	12.2 (8.8-16.0)	12.3 (9.0-16.7)
Total cholesterol (ref 3.0-6.7 mmol/l)	4.6 (3.9-5.5)	5.0 (4.1-5.9)

* For the subcohort of patients admitted between 2001 and 2006. Highest test result on day of intensive care unit (ICU) admission or the following day for creatinine, bilirubin C-reactive protein and white blood cell count and the lowest test results for hemoglobin. For cholesterol closets record value within one year before ICU admission.

IQR: interquartile range.

To assess possible unmeasured confounding by indication for statin treatment we restricted the analysis to patients with a previous diagnosis of ischemic or unspecified stroke, atherosclerosis, ischemic heart disease or diabetes mellitus. We also repeated the analysis after excluding patients with cancer and for users of non-statin lipid-lowering agents. Details on ICD codes are provided in Additional file 1.

To further control for confounding we conducted a supplementary analysis using propensity score matching [41,42]. We generated a multivariable logistic regression model that predicted statin use among ICU patients based on the covariate profile listed in Tables 1 and 2, except laboratory data and mechanical ventilation and dialysis, and computed the propensity score for all ICU patients. We then matched each statin user with one non-user using a greedy matching algorithm. All statin users could be matched to a non-user. Propensity score matching decreased the absolute standardized differences of each covariate to values below 0.1 indicating that an adequate balance was achieved. We then used Cox regression analysis to compute 30-day and one-year MRR in the matched cohort.

The assumptions of proportional hazards in all Cox regression models were assessed graphically and found to be appropriate. All analyses were performed using SAS version 9.2 (SAS Institute Inc, Cary, NC, USA).

The study was approved by the Danish Data Protection Agency (record number 2005-41-4782) and the Aarhus University Hospital Registry Board. Data were obtained from Danish registries, which are generally available to researchers and their use does not require informed consent.

## Results

### Descriptive data

We identified 12,483 first-time ICU patients older than 45 years. Of these, 1,882 (15.1%) were current statin users on admission (Table 1). Simvastatin was by far the most used statin (1391, 73.9%), followed by atorvastin (240, 12.8%), pravastatin (170, 9.0%) and other statins (81, 4.3%). Statin users were more likely to be male, and have higher levels of comorbidity than other ICU patients. Diabetes and cardiovascular diseases were, as expected, more prevalent among statin users than among non-users, whereas cancer and alcoholism-related diseases were less common. Statin users were more frequently users of ACE inhibitors, beta blockers or low-dose aspirin than non-users (Table 2). At ICU admission, statin users had higher average blood levels of creatinine, similar WBC count and slightly lower levels of CRP than non-users.

A total of 151 (8.0%) statin users and 917 (8.7%) non-users were hospitalized for more than 25 days during the 125 days before ICU admission.

### 30-day mortality

**Figure 1:**
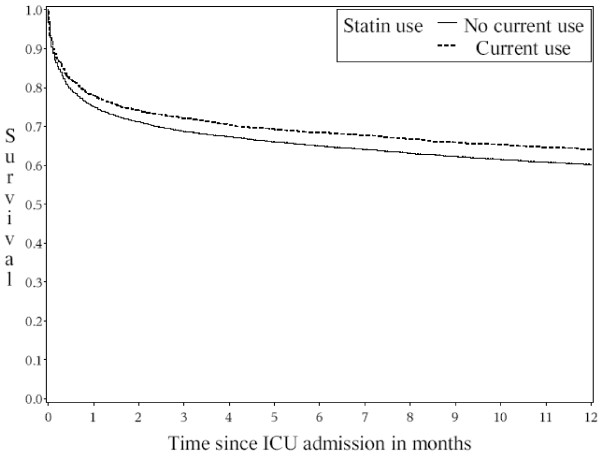
**One-year survival curve of 1881 statin users and 10,601 non-users**. ICU, intensive care unit.

**Figure 2:**
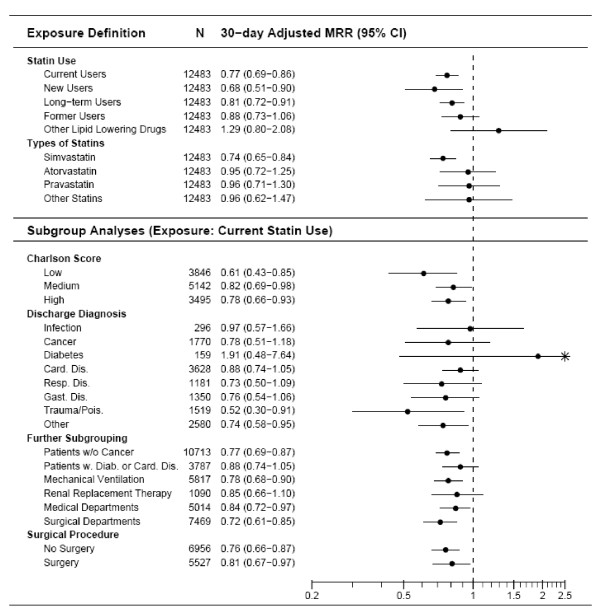
**Adjusted 30-day MRRs comparing preadmission statin use with non-use overall and within different patient subgroups**. N denotes the number of patients included in the analysis. CI, confidence interval; MMR, mortality rate ratios.

Throughout the follow-up period, statin users had considerably lower risk of death than statin non-users (Figure 1). The risk of death within 30 days after ICU admission was 22.1% among statin users and 25.0% among non-users, corresponding to a crude MRR of 0.85 (95% confidence interval (CI) = 0.76 to 0.96; Table 3). After control for potential confounders the 30-day MRR was 0.76 (95% CI = 0.68 to 0.85). For all diagnostic categories, except diabetes and infectious diseases, statin use was associated with a reduced risk of death (Figure 2). The MRRs seemed lower for users of simvastatin (MRR = 0.74, 95% CI = 0.65 to 0.84) than for other types of statins (atorvastatin MRR = 0.95, 95% CI = 0.72 to 1.25; pravastatin MRR = 0.96, 95% CI = 0.71 to 1.30); however, the differences were not statistically significant (*P *= 0.42).

**Table 3:** Cumulative 0 to 30 and 31 to 365 day mortality and corresponding crude and adjusted mortality rate ratios (MRR)

	Number (n)	Mortality (%)	Crude MRR (95% CI)	Adjusted MRR (95% CI)*
***0 to 30 days***				
Statin use	1882	22.1%	0.85 (0.76-0.96)	0.76 (0.68-0.85)
No statin use	11,313	25.0%	1 --	1 --
***0 to 365 days***				
Statin use	1882	36.4%	0.84 (0.76-0.93)	0.78 (0.71-0.84)
No statin use	11,313	39.9%	1 --	1 --

* Adjusted by Cox proportional hazards for age group, gender, medical/surgical department, diagnosis, Charlson index score and alcoholism-related disease, surgery within seven days, current use of angiotensin converting enzyme inhibitors, beta blockers and low-dose aspirin and marital status.

CI: confidence interval.

Exclusion of patients with cancer from the analysis or restriction to patients with cardiovascular diseases or diabetes left the decreased MRRs for statins virtually unchanged. Of note the risk of death seemed not to be reduced by non-statin lipid-lowering drug use (MRR = 1.29, 95% CI = 0.80 to 2.08) or by former statin use (MRR = 0.88, 95% CI = 0.73 to 1.06). For new and long-term statin users the adjusted MRRs were 0.68 (95% CI = 0.51 to 0.90) and 0.81 (95% CI = 0.72 to 0.91), respectively.

Further stratified analyses showed an adjusted 30-day MRR of 0.81 (95% CI = 0.67 to 0.97) among ICU patients who had surgery within seven days of ICU admission, and 0.76 (95% CI = 0.66 to 0.87) among ICU patients who had no surgery within seven days.

The propensity score matched analysis yielded a MRR similar to the estimates from the conventional Cox regression analysis (adjusted MRR = 0.71, 95% CI = 0.61 to 0.83).

### One-year mortality

**Figure 3:**
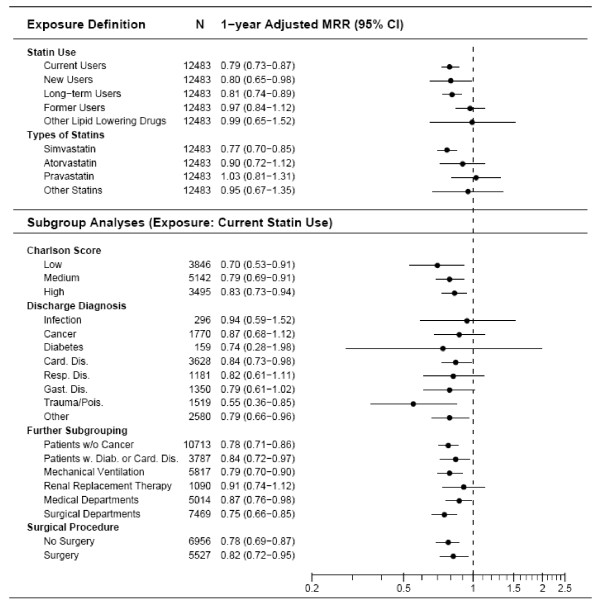
**Adjusted one-year MRRs comparing preadmission statin use with non-use overall and within different patient subgroups**. N denotes the number of patients included in the analysis. CI, confidence interval; MMR, mortality rate ratios.

The one-year risk of death was 36.4% among statin users compared with 39.9% among non-users; crude MRR was 0.84 (95% CI = 0.76 to 0.93) decreasing to 0.79 (95% CI = 0.73 to 0.87) after controlling for confounding factors. The one-year reduction in risk of death remained robust in all diagnostic categories and seemed most pronounced for simvastatin (simvastatin MRR = 0.77, 95% CI = 0.70 to 0.85; atorvastatin MRR = 0.90, 95% CI = 0.72 to 1.12; pravastatin MRR = 1.03, 95% CI = 0.81 to 1.31); however, the difference between statins did not reach statistical significance (*P *= 0.35). The reduction in risk of death associated with statin use remained virtually unchanged when analyses were restricted to patients without cancer or to patients with cardiovascular disease or diabetes (Figure 3). MRR among new and long-term statin users were 0.80 (95% CI = 0.65 to 0.98) and 0.81 (95% CI = 0.74 to 0.89), respectively. Former statin use and use of non-statin lipid-lowering drugs was not to be associated with reduced risk of death within one year of ICU admission.

The propensity score matched analysis yielded an adjusted MRR of 0.70 (95% CI = 0.62 to 0.80).

## Discussion

In this large cohort study we found that preadmission statin use was associated with considerably reduced risk of death among ICU patients. The reduced risk of death remained robust in various subgroup analyses, including among new and long-term statin users. We found no clear association between former statin use and non-statin lipid-lowering drug use and risk of death, which supports a causal association between statin use and reduced risk of death among ICU patients.

### Existing studies

Increasing evidence exists that statins may reduce mortality in patients with severe infections including sepsis [13-17] but limited and conflicting data exist on the association between statin use and in-hospital/30-day mortality among general ICU patients. In a 2006 German cohort study of 120 ICU patients with multiple organ dysfunction syndrome, Schmidt and colleagues reported that statin use was associated with substantially reduced in-hospital mortality (MRR = 0.53, 95% CI = 0.29 to 0.99), a result consistent with our findings [25]. Also in line with our findings, a recent US study among 178 patients with ALI/ARDS found reduced ICU and in-hospital mortality among statin users (odds ratio (OR) for ICU mortality 0.82, 95% CI = 0.36 to 1.89; OR for in-hospital mortality 0.62, 95% CI = 0.29 to 1.32); however, the relatively small study population hindered a clear interpretation of the risk estimates [26]. In contrast, a 2006 Spanish observational study of 438 patients mechanically ventilated for more than 96 hours reported higher in-hospital mortality among statin users (61%) than non-users (42%; OR = 2.30, 95% CI = 1.08 to 4.89) [24]. All studies included highly selected subgroups of ICU patients and did not adjust for important covariates such as comorbidity and use of other cardiovascular drugs. To our knowledge, no former data exist on the effect of statins on long-term risk of death among ICU patients.

The biological mechanisms underlying our observations are not yet entirely understood. However, the high prevalence of systemic inflammatory response syndrome and/or severe infections among ICU patients may increase the risk of fatal venous and arterial thrombotic events by inducing endothelial dysfunction and atherosclerotic plaque instability [43-45]. There is strong experimental evidence that statins have beneficial effects on platelet function, coagulation, fibrinolysis, and plaque formation, as well as inhibitory effects on endothelial dysfunction [46-50]. Also, statins have immuno-modulating effects that may be beneficial during the initial 'hyper-immune' phase of critical illness [5,23]. Differences in these pleiotropic properties between lipophilic and hydrophilic statins may explain the possible differences between individual statins suggested by our data, although these differences were not statistically significant [7-9]. One randomized controlled trial including 82 patients with acute bacterial infections confirmed that statins reduce the levels of inflammatory cytokines [51].

### Limitations

We conducted this study within a tax-financed national health service system with free access to health care, which largely removed referral bias. We had complete and independently collected data on preadmission statin use and one-year mortality, which limited the risk of information and surveillance bias. The relatively large number of ICU patients enabled robust analysis on several ICU subgroups showing consistent results.

The validity of our findings depends ultimately on accurate registration of statin use and the ability to control for confounding. The completeness and nature of the prescription database used makes the measurement of filled prescriptions for statins highly valid [31]. In Denmark, adherence to statin therapy is high, and any influence of non-compliance should therefore be minor, potentially attenuating our mortality estimates towards unity [52]. As few statin users were hospitalized for more than 25 days during the 125 days before ICU admission, misclassification of statin use because of more frequent previous hospitalizations most likely had little influence on our results [33]. We had access to data on a large number of prospectively collected covariates from databases with high validity for surgical procedures, diagnosis codes, and laboratory data. Still, any lack of specificity in routinely recorded data may have reduced our ability to completely remove confounding and most likely would attenuate our findings towards the null. Severe confounding by socioeconomic differences between statin users and non-users is unlikely given the tax-financed Danish public health care system [53,54]. This is supported by a recent study that found similar reduced risk of all-cause mortality among statin users and non-users when comparing results from the randomised 4S study with results from an observational study based on prescription data [55].

Statins may exert their potential beneficial effects by improving immune dysfunction caused by critical illness. Surrogate physiological measures of severity of illness and inflammation, e.g. CRP and WBC count, may thus be in the pathway between statin use and mortality and do not fulfil the criteria for being confounding factors [40,55]. We therefore did not adjust for physiological measures, including laboratory data, in the analysis.

## Conclusions

In this large cohort study, preadmission use of statins was associated with reduced risk of death within 30 days and one year in general ICU patients. The associations seen could be a pharmacological effect of statin use; however, it remains to be fully clarified whether differences in characteristics of statin users and non-users may explain at least part of the associations found.

## Key messages

• In experimental studies statins have shown to have anti-inflammatory, anti-thrombotic and immuno-modulating effects independent of lowering lipids, which may reduce mortality from critical illness.

• In observational studies statin use has been associated with reduced mortality following major surgery and severe infections.

• We found that preadmission statin use is associated with reduced short- and long-term mortality following intensive care.

• The beneficial effects may be most pronounced for users of simvastatin.

## Abbreviations

ACE: angiotensin converting enzyme; ALI/ARDS: acute lung injuri/acute respiratory distress syndrome; ATC: Anatomical Therapeutic Chemical; AUICC: Aarhus University Intensive Care Cohort; CRP: C-reactive protein; ICD International Classification of Diseases; ICU: intensive care unit; MRR: mortality rate ratio; NRP: National Register of Patients; WBC: white blood cell.

## Competing interests

The authors declare that they have no competing interests.

## Authors' contributions

SC, RWT, AL, ET and HTS conceived the study idea. SC and HTS designed the study. RJ and KML collected the data. SC, HTS, MBJ and LP analysed the data. All authors interpreted the findings. SC and RWT reviewed the literature. SC wrote the first draft and all authors edited the manuscript and approved the final version.

## Supplementary Material

Additional file 1 **International Classification of Diseases (ICD) and Anatomical Therapeutic Chemical (ATC) codes**. Word document containing ICD and ATC codes for diagnoses and drugs included in the analyses of the current study. Click here for file
