# Supplementary material for: Preadmission statin use and one-year mortality among patients in intensive care - A cohort study
Source: Crit Care. 2010 Mar 9;14(2):R29. doi: 10.1186/cc8902 (PMC2887131; doi:10.1186/cc8902)
Supplement: Additional file 1 — International Classification of Diseases (ICD) and Anatomical Therapeutic Chemical (ATC) codes. Word document containing ICD and ATC codes for diagnoses and drugs included in the analyses of the current study. [file cc8902-S1.DOC]

**Additional file 1: ICD and ATC Codes**

Disease categories

Infectious diseases (ICD-10:A00-B99), endocrinology including diabetes (ICD-10:E00-E90), cardiovascular diseases (ICD-10: I00-I99), respiratory diseases (ICD-10: J00-J99), gastrointestinal and liver disease (ICD-10: K00-K99), cancer (ICD-10:C00-D89), trauma and poisoning (ICD-10: S00-T98) and others (ICD-10: all codes not included in other categories)

Alcoholism-related disorders

ICD-8: 291, 303, 979, 980,571.09, 571.10, 577.10

ICD-10: G62.1, G72.1, G31.2, I42.6, F10, K29.2, K70, K86.0, Z72.1, R78.0, T51

AND/OR

Previous prescriptions of disulfiram (ATC: N07BB01)

Ischemic or unspecified stroke

ICD-8: 433-434; ICD-10: I63-I64, I69.3, I69.4, I69.8

Atherosclerosis

ICD-8: 440, ICD-10: I70

Ischemic heart disease

ICD-8: 410-440, ICD-10: I20-I25

Diabetes

ICD-8: 249-250, ICD-10: E10-E11

Cancer

ICD-8:140-194, 195-198, 199, 200-203, 204-207, 275.59;

ICD-10: C00-C75, C76-C80, C81-C85, C88, C90, C91-C95, C96

Medications (ATC codes)

Simvastatin: C10AA01, B04AB01

Atorvastatin: C10AA05, B04AB05

Pravastatin: C10AA03, B04AB03

Other statins: C10AA0X, B04AB0X, not included in other categories.

ACE-inhibitors: C09

Beta-blockers: C07

Low-dose aspirin: N02BA01

Non-statin lipid lowering drugs [niacin, bile acid binding resin and fibric acid derivatives]: C10AD
